# ORTHOPEDIC INJURIES IN SOCCER - AN ANALYSIS OF A PROFESSIONAL CHAMPIONSHIP TOURNAMENT IN BRAZIL

**DOI:** 10.1590/1413-785220172505171247

**Published:** 2017

**Authors:** RAFAEL FONSECA RODRIGUES DE SOUZA, SERGIO MAININE, FABIANO FONSECA RODRIGUES DE SOUZA, ENRICO MONTORSI ZANON, ALEXANDRE YUKIO NISHIMI, EIFFEL TSUYOSHI DOBASHI, FÁBIO ARAÚJO FERNANDES

**Affiliations:** 1. Department of Orthopedics and Traumatology, Hospital IFOR- Rede D´Or, São Bernardo do Campo, SP, Brazil.

**Keywords:** Soccer, Athletic injuries, Wounds and injuries

## Abstract

**Objective:**

To analyze the incidence of orthopedic injuries which occurred during a professional soccer championship in São Paulo, Brazil in 2010.

**Methods:**

This assessment collected data from the pre-season until the final stage of the championship.

**Results:**

We analyzed 227 professional players from eight of the top teams in this championship. Data were obtained for 71.02% of all games. The athletes were all male with a mean age of 23.1 years; the average number of injuries was 1.6 per athlete, with muscle injuries and sprains resulting from indirect origin predominating in the legs.

**Conclusion:**

Injuries were more frequent in forwards and outside backs, and players generally returned to play within one week of treatment. **Level of Evidence III, Study of Non Consecutive Patients; Without Consistently Applied Reference “Gold” Standard.**

## RESUMO

**Objetivo:**

Analisar a incidência de lesões ortopédicas ocorridas durante o Campeonato Paulista Profissional da Série A2 de 2010.

**Métodos:**

Esta avaliação restringiu-se à coleta de dados desde a pré-temporada até a fase final do campeonato.

**Resultados:**

Foram analisados 227 jogadores profissionais de oito das principais equipes deste campeonato. Foram obtidos dados de 71,02% do total de jogos. Os atletas eram todos do sexo masculino, com média de idade de 23,1 anos; o número médio de lesões foi de 1,6 por atleta com predomínio de lesões musculares e entorses indiretos nos membros inferiores.

**Conclusão:**

Houve uma frequência maior de lesão em atacantes e laterais, com retorno predominante ao esporte em até uma semana de tratamento. **Nível de Evidência III, Estudo de Pacientes Não Consecutivos; Sem Padrão de Referência “Ouro” Aplicado Uniformemente.**

## INTRODUCTION

Competitive sports became more popular during the nineteenth century, beginning with the Modern Olympic Games in Athens and Greece in 1896. The populations of many countries were encouraged to exhibit their sports performances and seek superiority. Many sports were created and developed, and some reached great popularity; most notable among these is soccer, which is among the most popular sports practiced by both sexes in different age groups. FIFA, the International Football Federation, currently contains 203 member countries and approximately 200 million players.^1^

In Brazil, the history of soccer begins with Charles Miller, who was born in the Brás neighborhood of São Paulo. When he was nine years old, he went to England to study and came into contact with soccer; when he returned to Brazil in 1894 he brought the first soccer ball and a set of rules. Brazil’s first soccer game took place on April 15, 1895 between employees of British companies which were present in São Paulo. The first team to form in Brazil was São Paulo Athletic, founded on May 12, 1888. Initially, only the elites played soccer, and Blacks were even banned from teams, but over time drastic changes took place in the sport, mainly due to professionalism and the increasing physical demands which forced athletes to work to near-exhaustion and caused them to be more predisposed to injuries.^2^

In our environment, it has been difficult to achieve a balance between preparation and athletic demands. Advances in sports medicine have led us to a better understanding of the physiology of effort, allowing specific protocols for each athlete and individualizing their characteristic; on the other hand, the large number of games, trainings, and the broad availability of athletes, places them at risk for injuries to muscles, bones, and joints.^3^

Athletes are considered to be models of health because of their optimal physical capacity, and therefore have trouble accepting the need to trade the soccer field for the medical department.^3^ In some situations, like injuries in professional athletes, physicians should be fully aware of their own behavior, because in soccer there is constant pressure to keep players on the field or to return players to action as early as possible.

Historically, in 1952 Naves^4^ was the first to report a relationship between soccer and traumatic injuries over 4,000 games and 10 years. In 1978, McMaster and Walter^5^ performed the first prospective study of soccer injuries in the American literature. Also in 1978, Nilsson and Roaas^6^ studied two Norwegian championships and found that 56% of injuries occurred during the period of the game, two-thirds of these affected the legs, and that contusions were the most common injuries. In 1989, Ekstrand and Nigg^7^ associated soccer injuries with the use of inadequate footwear as well as the type of grass or soil used. 

In the Brazilian literature, in 1992 Carazzato et al.^8^ studied and compared field and indoor soccer injuries according to diagnosis and anatomic location of injuries. And in 1994, Pedrinelli^9^ surveyed 354 traumatic injuries in 150 professional soccer players, highlighting age, injury location, the athlete’s position on the field, and etiology. This researcher concluded that the most frequent injuries occurred in the legs, without contact.

There are many variables related to injuries in soccer, so we divided these variables into two groups: 1) intrinsic - those which are inherent to the sport itself, such as short and fast runs, leaps, quick changes in direction, heading the ball, etc.; and 2) extrinsic - which evaluate the field conditions, type of footwear, physical conditions and health, sex, number of games, training, and motivation.

Considering the characteristics of soccer, the objective of this study was to analyze the incidence of orthopedic injuries which occurred during the A2 series of the 2010 Campeonato Paulista Profissional (São Paulo Professional Championship).

## MATERIALS AND METHODS

In this study, we analyzed the medical records of 227 professional players from 8 of the top teams of the A2 series of the 2010 Campeonato Paulista professional soccer championship. This assessment was restricted to data collected from the pre-season until the final stage of the championship.

The inclusion criterion was professional soccer players who competed in the A2 series of the 2010 Campeonato Paulista. The exclusion criterion was loss to follow-up of athletes after leaving the club or the sport or for some other reason that did not permit us to analyze the time it took to return to the sport.

The variables used in this study were: number of players available for the competition, total number of games, age, position played, mechanism of injury, anatomical location of the injury, type of injury, temporality of the injury (pre-season, training, or game), treatment established, time lost from play, and conditions to return to the sport.

The injuries were assessed according to each player’s position on the field, namely goalkeeper, outside back (*lateral*), central defender (*zagueiro*), midfielder, center forward (*centroavante*), and right/left forwards (*ponta*).

The diagnoses were made by the team physicians and divided into contusions, fractures and dislocations, ligament injuries, muscle injuries, and tendonitis. Complementary exams were used for follow-up, such as chest x-rays, ultrasound, and magnetic resonance imaging.

Injury locations were classified by segments: trunk (head and neck, dorsal spine and thorax, lumbar spine and pelvic girdle), legs (thigh, knee, leg, ankle, and foot), and arms (shoulder, arm, elbow, forearm, wrist, and hand).

The injuries were classified according to the mechanism of occurrence, direct contact and non-contact.

The period of time lost was considered to span the time of injury until release to participate in team practice, and used to classify the injuries as mild (0-7 days), moderate (8-30 days), or severe (> 31 days). 

The data were obtained from the athletes’ medical records, which in turn were obtained from their teams. These variables were recorded on spreadsheets which were computed and analyzed by the authors of this study.

The championship competition, which included 20 participating teams, ran from January 13 to May 2, 2010 and was conducted in two stages in which each team played at least 19 and at most 25 games. During the first stage, the 20 teams played against each other once in order to classify for the second stage, in which the 8 teams with the highest point scores played. These 8 teams were divided into two groups of 4 which played among their respective groups in a match and rematch.

The pre-season period, which immediately precedes the championship, lasted an average of 30 days and required the medical department professionals to dedicate more time to their team for planning and implementing specific tasks for the start of the season. As many athletes return from vacations or downtime, the rigor of the beginning of the season implies demands on the musculoskeletal system that can generate injuries from overload. The pre-season allows physicians and physiotherapists to see which athletes will require preventive care during the season to withstand their game calendar, which generally is intense and irregular.

Foot injuries such as blisters and calluses are frequent and less severe, and may even harm performance in team practice and physical training sessions, but these were not analyzed due to their lack of correlation with orthopedic injuries.

The average practice time followed a theoretical standard composed of 10 to 14 training periods per week (two three-hour training periods per day); twice a week, before and after games, the athletes were gathered but did not perform physical activities. Changes were made when necessary according to team needs.

## RESULTS

The championship had a total of 214 games and 7,436 fouls, averaging 34.0 fouls per game: 1,265 yellow cards, an average of 5.0 per game, and 141 red cards with an average of 0.65 per game. (Table 1)

**Table 1:** General data per round of the A2 series of the Campeonato Paulista professional football championship.

	Games		Fouls		Goals		Yellow Cards		Red Cards		Game Play	
	Games	Total	Fouls	Mean	Goals	Mean	Yellow	Mean	Red	Mean	Time	Mean
Round 01	1 0	10	345	34.00	25	200	52	5.00	2	0.00	10:03:27	01:00:20
Round 02	10	20	409	40.00	23	200	55	5.00	5	0.00	09:55:21	00:59:32
Round 03	10	30	355	35.00	14	1.00	60	6.00	5	0.00	10:15:04	01:01:30
Round 04	10	40	380	38.00	35	3.00	54	5.00	5	0.00	10:08:08	01:00:48
Round 05	10	50	370	37.00	31	3.00	71	7.00	9	0.00	09:58:08	00:59:48
Round 06	10	60	312	31.00	33	3.00	61	6.00	3	0.00	10:10:46	01:01:04
Round 07	10	70	377	37.00	33	3.00	64	6.00	3	0.00	10:16:00	01:01:36
Round 08	10	80	310	31.00	20	2.00	69	6.00	10	1.00	09:56:26	00:59:38
Round 09	10	90	338	33.00	27	2.00	45	4.00	2	0.00	10:10:55	01:01:05
Round 10	10	100	346	34.00	39	3.00	59	5.00	8	0.00	10:12:06	01:01:12
Round 11	10	110	318	31.00	31	3.00	50	500	6	0.00	10:10:29	01:01:02
Round 12	10	120	364	36.00	31	3.00	68	6.00	12	1.00	09:55:44	00:59:34
Round 13	10	130	342	34.00	30	3.00	56	5.00	7	0.00	09:58:00	00:59:48
Round 14	10	140	368	36.00	28	2.00	58	5.00	12	1.00	09:58:30	00:59:51
Round 15	10	150	350	35.00	39	3.00	57	5.00	5	0.00	10:23:45	01:02:22
Round 16	10	180	332	33.00	35	3.00	65	6.00	11	1.00	10:14:45	01:01:28
Round 17	10	170	294	29.00	29	2.00	43	4.00	5	0.00	09:53:06	00:59:18
Round 18	10	180	359	35.00	33	3.00	83	8.00	6	0.00	10:09:29	01:00:56
Round 19	10	190	309	30.00	34	3.00	53	5.00	8	0.00	10:05:41	01:00:34
Round 20	4	194	135	33.00	11	2.00	21	5.00	4	1.00	03:58:40	00:59:40
Round 21	4	198	165	41.00	12	3.00	21	5.00	5	1.00	04:09:25	01:02:21
Round 22	4	202	142	35.00	8	1.00	24	6.00	1	0.00	04:03:00	01:00:45
Round 23	4	206	158	39.00	14	3.00	28	7.00	3	0.00	04:08:45	01:02:11
Round 24	4	210	133	33.00	12	3.00	25	6.00	1	0.00	03:59:59	00:59:59
Round 25	4	214	125	31.00	19	4.00	23	5.00	3	0.00	04:07:00	01:01:45
Championship total		214	7.438	34.00	644	3.00	1.265	5.00	141	0.00	16:22:39	01:00:39

Source: Paulista Soccer Federation.

Of the 227 medical records, a total of 42 athletes were excluded from our sample. Data were obtained from 152 of the 214 games during this championship, for a total of 71.02%, but data were analyzed for 40%, for 8 of the 20 participating teams. Each team had between 26 and 38 players available, with an average of 28.4 athletes per team. Player ages ranged from 17 to 42 years, with a mean of 23.1. 

**Figure 1:**
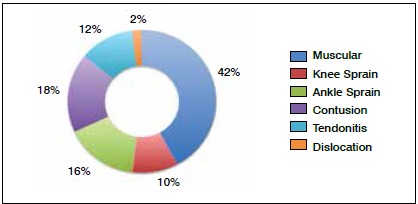
Types of injuries presented per athlete.

The average number of orthopedic injuries per athlete was 1.6 in 4 months. Figure 1 shows the types of injuries sustained by the athletes, with a predominance of muscle injuries and sprains.

**Figure 2:**
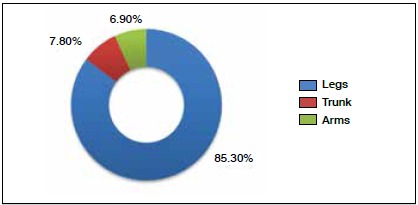
Anatomic location of injuries.

**Figure 3:**
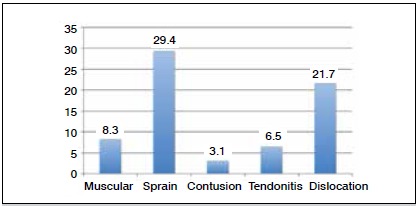
Average time before returning to play, in days.

As for anatomic location, 85.3% (n = 309) involved the legs, 7.8% (n = 28) were in the trunk, and 6.9% (n = 25) the arms. (Figure 2) The vast majority of injuries were diagnosed clinically. Only in the more serious cases in which doubt was present were the athletes were subjected to complementary examinations. The average time lost as a result of injury varied markedly according to injury type, as can be seen in Figure 3, but in some cases the player was lost to follow-up after injury.

**Figure 4:**
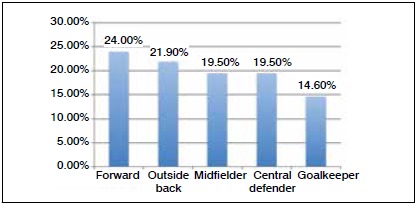
Average number of injuries according to position.

Figure 4 shows the average number of injuries according to the position of the athlete. In our sample, the forward players and outside backs had the most injuries during the season.

**Figure 5:**
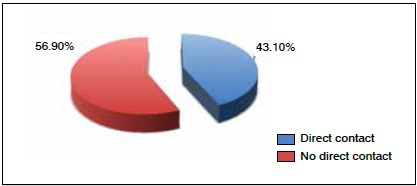
Mechanism of injury.

We found 43.1% of injuries resulted from direct contact and 56.9% involved no contact. (Figure 5) Of the non-contact injuries, 62.1% were muscle injuries, while 38.9% of injuries from direct contact were contusions.

## DISCUSSION

The analysis of the number of injuries according to player position showed a lower incidence in goalkeepers. When comparing defensive and offensive players, we observed a clear predominance of injuries in forwards and outside backs according to numbers alone; however, we must bear in mind the total number of athletes available for each position and the degree of trauma exposure to which each athlete is subjected.

As for frequency of injury diagnoses, our sample presented data compatible with those in the national literature by Ejnisman and Cohen^1^ with a greater number of muscle injuries, but conflicted with the sample studied by Nilsson and Roaas,^6^ who found a higher incidence of contusions. 

We must bear in mind that many athletes ignore their injuries, self-medicate, and seek guidance from professionals who are unrelated to their team medical department for fear that they will be benched, or as a result of lack of knowledge or greater confidence in another professional.

The lost time was greater for dislocations and fractures than other types of injury. The vast majority of contusions and sprains did not prevent the athletes from returning to football after more than a week, despite their high prevalence. Time lost to play did not follow a homogeneous pattern; some athletes relapsed when they returned to the game.

Because of the highly competitive of this soccer league, in our sample some players were lost to follow-up after injuries, transfers, being released from the team, or leaving the sport, among other situations. This occurred in most of the teams analyzed, which prevented us from accurately ascertaining physical response after returning to professional play. In some cases, the team was completely restructured due to their position in the rankings and hired new players during the championship, causing an information bias due to the total time the athlete was followed.

We found no studies in the literature with results comparable to those presented herein.

This is an observational, cross-sectional study containing information that reflects the specific analysis of this tournament. Other studies should be conducted to compare athletes in other championships, in several categories, to confirm possible differences in the results. 

Consequently, efforts should be made in the area of physical preparation as well as the medical area so that mechanisms of injury prevention can be successfully implemented in professional soccer.

## CONCLUSION

Based on the data from this study, we concluded that the frequency of injuries in professional athletes in football in a season was extremely high, since 61% of the athletes had some kind of injury during a season. The forward players and outside backs are most affected by injuries, predominantly via indirect trauma; muscle injuries were the most prevalent and legs were the most affected area of the body. 
